# Transcriptome analysis reveals *FABP5* as a key player in the development of chicken abdominal fat, regulated by miR-122-5p targeting

**DOI:** 10.1186/s12864-023-09476-1

**Published:** 2023-07-10

**Authors:** Bin Zhai, Hongtai Li, Shuaihao Li, Jinxing Gu, Hongyuan Zhang, Yanhua Zhang, Hong Li, Yadong Tian, Guoxi Li, Yongcai Wang

**Affiliations:** 1 College of Animal Science and Technology, Henan Agricultural University, Zhengzhou, 450046 China; 2 The Shennong Laboratory, Zhengzhou, 450046 China; 3 Henan Key Laboratory for Innovation and Utilization of Chicken Germplasm Resources, Zhengzhou, 450046 P. R. China

**Keywords:** RNA-seq, miR-122-5p, *FABP5*, Chicken abdominal fat, Differentiation

## Abstract

**Background:**

The development of abdominal fat and meat quality are closely related and can impact economic efficiency. In this study, we conducted transcriptome sequencing of the abdominal fat tissue of Gushi chickens at 6, 14, 22, and 30 weeks, and selected key miRNA-mRNA regulatory networks related to abdominal fat development through correlation analysis.

**Results:**

A total of 1893 differentially expressed genes were identified. Time series analysis indicated that at around 6 weeks, the development of chicken abdominal fat was extensively regulated by the TGF-β signaling pathway, Wnt signaling pathway, and PPAR signaling pathway. However, at 30 weeks of age, the apoptosis signaling pathway was the most significant, and correlation analysis revealed several genes highly correlated with abdominal fat development, including Fatty Acid Binding Protein 5 (*FABP5*). Based on miRNA transcriptome data, it was discovered that miR-122-5p is a potential target miRNA for *FABP5*. Cell experiments showed that miR-122-5p can directly target *FABP5* to promote the differentiation of preadipocytes.

**Conclusion:**

The present study confirms that the key gene *FABP5* and its target gene miR-122-5p are critical regulatory factors in the development of chicken abdominal fat. These results provide new insights into the molecular regulatory mechanisms associated with the development of abdomen-al fat in chickens.

**Supplementary Information:**

The online version contains supplementary material available at 10.1186/s12864-023-09476-1.

## Background

In recent years, the selective breeding of poultry has resulted in rapid growth rates in chickens. This accelerated growth leads to excessive fat deposition, particularly in the abdominal region, which in turn has negative impacts on feed efficiency, egg production, fertilization, and hatchability [1–3]. Consequently, molecular mechanisms behind abdominal fat deposition have been a hot topic of research in the fields of avian genetics and breeding. However, adipose tissue development is an exceedingly complex process which involves, on the one hand, the proliferation of precursor adipocytes and their differentiation into mature adipocytes, and on the other hand, the proliferation of adipocytes and accumulation of lipid droplets within these cells [4, 5]. Studies have shown that in chicken embryos, mesenchymal stem cells differentiate into preadipocytes, followed by their proliferation and eventual differentiation into mature adipocytes. Early development in chicken embryonic adipose tissue is characterized by the dominant proliferation of preadipocytes, which then differentiate into mature adipocytes under the control of several transcription factors and regulatory genes involved in adipogenesis [6–8]. At 12 days of incubation, a large number of preadipocytes can be observed in chicken embryos, while at 14 days, mature adipocytes are readily detectable; this period marks a rapid transition of preadipocytes into mature adipocytes [9]. As adipocyte differentiation proceeds, immature adipocytes accumulate and lipid droplet deposition begins during the adipocyte maturation stage. During the entire post-hatching growth process in chickens, adipocyte hypertrophy is concomitant with adipocyte hyperplasia, which continues until 12–14 weeks of age [10]. Therefore, abdominal fat deposition in chickens primarily proceeds through three distinct stages: proliferation of preadipocytes, differentiation of preadipocytes into adipocytes, and accumulation of lipid droplets in mature adipocytes. RNA-seq analysis of NanDanYao chickens, separated into high and low fat groups, revealed certain genes related to abdominal and muscular fat deposition [11]. Recently, transcriptome technology has been extensively used to investigate the development of chicken abdominal fat due to its ability to accurately measure the abundance of genes and transcripts. For instance, researchers have identified several differential genes associated with fat synthesis by comparing lean and fat chicken strains[12]. Nevertheless, these studies have mainly focused on the differences in fat deposition between different or within the same strains, neglecting the fact that the development of fat is an extremely complex and continuous process. Therefore, it is imperative to uncover the key genes and regulatory networks involved in the development of chicken abdominal fat.

Fatty acid binding proteins (FABPs) were a family of small, highly conserved cytoplasmic proteins whose effects include fatty acid uptake, transport and metabolism [13]. *FABP5* was a member of the fatty acid binding proteins family. Several studies have found that *FABP5* was involved in the process of several cancers through metabolic reprogramming [13–16]. *FABP5* was significantly downregulated in chicken intramuscular fat compared to abdominal preadipocytes [17]. A previous study has shown that *FABP5* is highly correlated with triglyceride (TG) content [18]. MicroRNAs (MiRNAs) were a group of evolutionarily conserved endogenous miRNAs, approximately 22 nucleotides in length, that play a pivotal role in regulating fat production[19–21]. MicroRNA-122 was one of the most abundant miRNAs in the liver, and it was also expressed in fat, heart, skeletal muscle and other tissues [22]. The miR-122 has important roles in liver growth and development, lipogenesis as well as in liver diseases [22, 23]. However, it was currently unclear whether there was a regulatory relationship between *FABP5* and miR-122 in regulating the deposition of abdominal fat in chickens.

Gushi chickens, as a renowned indigenous breed in China, are celebrated for their delicate meat texture, distinctive flavor, and abundant nutritional qualities. It is frequently utilized as breeding material due to these exceptional attributes. The excessive accumulation of abdominal fat in Gushi hens during the production process has consistently yielded negative consequences, leading to a decline in their reproductive performance. However, the understanding of the key genes and signaling pathways that play pivotal roles in abdominal fat development at different physiological stages in Gushi chickens, as well as the miRNA post-transcriptional regulatory network, remains elusive. Therefore, in this study, we performed high-throughput sequencing on abdominal fat tissue samples obtained from Gushi chickens at 6, 14, 22, and 30 weeks of age, with a specific focus on identifying the key genes involved in abdominal fat development during different stages of Gushi chicken growth and constructing their miRNA post-transcriptional regulatory network. The obtained results provide a foundation for further studying the molecular mechanisms underlying the regulation of abdominal fat deposition in chickens.

## Results

### Gene expression Profile related to Abdominal Fat Development

In this investigation, RNA-seq analysis was conducted on the abdominal adipose tissues of 12 Gushi chickens at four distinct developmental stages. Each library generated 93,729,158 to 116,860,112 raw reads, which underwent quality control using fastp. The clean bases in each library ranged from 13.53 to 16.88 GB, with the CG content ranging from 46.20 to 51.56%. Comparative analysis revealed that 91.97–95.55% of the reads (83.08–90.16% of the number of species) were located in the reference genome (Table S1). The transcripts sequenced from each library were merged using stringtie merge software to identify the characteristics of mRNA in each library, which were subsequently screened and annotated. A total of 16,693 known mRNA and 103 novel mRNA (218 transcripts) were identified from the 12 libraries.

**Fig. 1:**
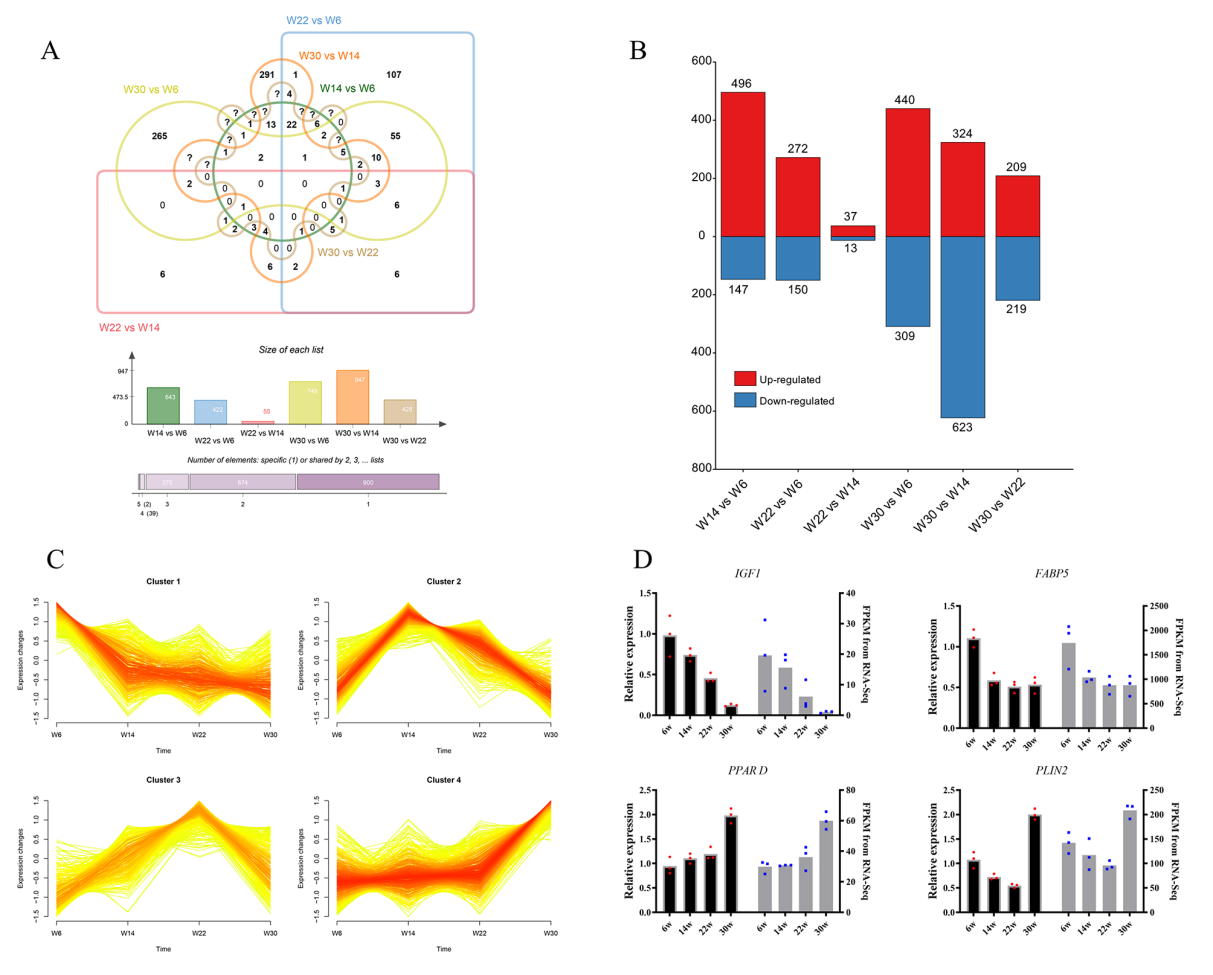
Expression patterns of genes associated with abdominal fat development in Gushi chickens and validation of sequencing data. (**A**) Venn diagram of the DE-mRNAs. (**B**) Column chart illustrating differentially upregulated and downregulated genes in each comparison group, red color represents upregulation, while blue color represents downregulation. (**C**) Mfuzz clustering results of differentially expressed mRNA in abdominal fat development of Gushi chicken. (D) The qRT-PCR verification of DE-mRNA.

DEseq2 was employed for differential analysis (|fold change, FC|≥1.5, q-value < 0.05). Among the six comparison groups, namely W14 vs. W6, W22 vs. W6, W22 vs. W14, W30 vs. W6, W30 vs. W14, and W30 vs. W22, there were 643(496 up-regulated, 147 down-regulated), 422(272 up-regulated, 150 down-regulated), 50(37 up-regulated, 13 down-regulated), 749(440 up-regulated, 309 down-regulated), 947(324 up-regulated, 623 down-regulated), and 428(209 up-regulated, 219 down-regulated) DE-mRNAs, respectively (Fig. 1A,B). Mfuzz analysis results show that the expression patterns of 1,893 DE-mRNAs were classified into 4 profiles (Fig. 1C). Notably, cluster 2 and cluster 3 exhibited similar expression patterns, with cluster 2 displaying the highest expression at 14 weeks of age, and then declining, while cluster 3 had the highest expression at 22 weeks of age, and then declining as well. Meanwhile, cluster 1 showed a gradual decrease in expression over time, while cluster 4 showed an increase in expression with the progression of development. Moreover, four DE-mRNAs were randomly selected for qRT-PCR verification, and the outcomes were consistent with the expression pattern obtained by RNA-seq sequencing, signifying the authenticity and reliability of the RNA-seq sequencing data (Fig. 1D).

### Key genes and regulatory network related to abdominal fat development

**Fig. 2:**
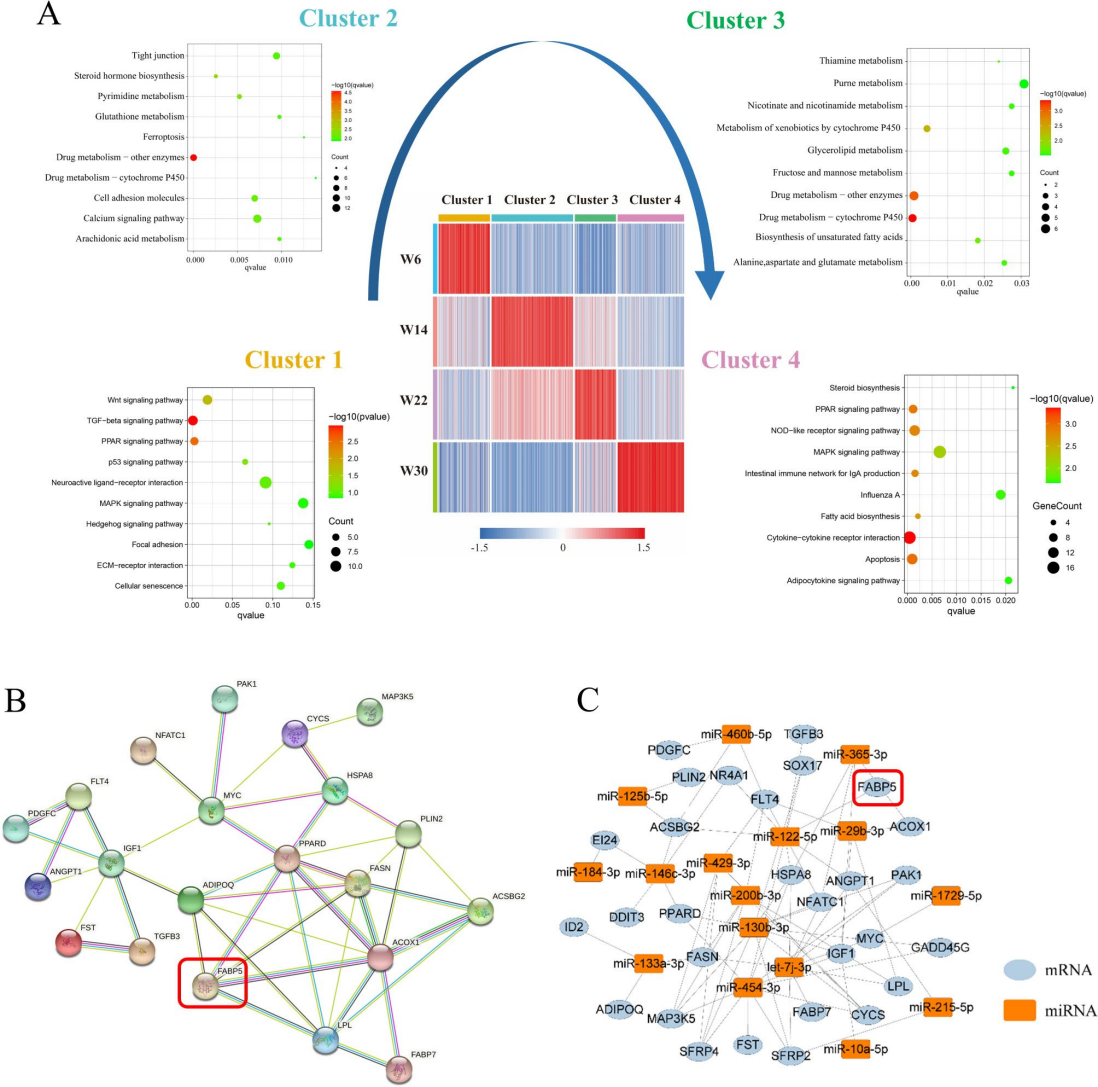
KEGG signaling pathway enrichment analysis of differentially expressed gene clusters (clusters 1–4) and regulatory network related to abdominal fat development. (**A**) Present a heatmap depicting the gene expression levels of the four clusters and exhibit the KEGG signal pathway analysis for the genes belonging to each cluster. (**B**) The protein-protein interaction network of genes related to abdominal fat development in Gushi chicken. (**C**) The miRNA-mRNA interaction network related to abdominal fat development in Gushi chicken, with mRNA in blue and miRNA in orange

We conducted functional enrichment analysis for each cluster. The GO enrichment results revealed that cluster 1 was significantly enriched in DNA packaging, regulation of gene silencing, negative regulation of gene expression, and epigenetics. On the other hand, clusters 2 and 3 were significantly enriched in GO terms such as cell junction, plasma membrane, overall composition of membrane, extracellular space, and extracellular region. Finally, cluster 4 was significantly enriched in GO terms such as receptor ligand activity, signal receptor activator activity, and receptor regulator activity (Figure S1). KEGG results revealed that cluster 1 was enriched in the TGF-β signaling pathway, PPAR signaling pathway, Wnt signaling pathway, and p53 signaling pathway; clusters 2 and 3 were enriched in several metabolism-related signaling pathways, including Drug metabolism - other enzymes, Drug metabolism - cytochrome P450, and Nucleotide metabolism; in contrast, cluster 4 showed enrichment in signaling pathways such as cytokine-cytokine receptor interaction, apoptosis, PPAR signaling pathway, NOD-like receptor signaling pathway, fatty acid biosynthesis, and more (Fig. 2A).

Through Mfuzz analysis, we analyzed the PPI network of genes related to abdominal fat development with the STRING 11.5 database. The results indicated the presence of multiple central nodes among these genes (Fig. 2B). There were two important nodes in the network, including *PPARD* and *IGF1*. *PPARD* can regulate 8 genes, including *LPL*, *ACOX1*, *ADIPOQ*, *FABP5*, *HSPA8*, *PLIN2*, *FASN*, and *MYC*. *IGF1* can regulate 7 genes, including *FLT4*, *ANGPT1*, *FST*, *TGFB3*, *MYC*, *PDGFC*, and *ADIPOQ*. Regarding these hub nodes, this suggests that they may play essential roles in the complex physiological processes of abdominal fat development. Additionally, using miRNA transcriptome profile data from abdominal fat samples of Gushi chickens at four developmental stages, we identified 51 differentially expressed miRNAs[24]. By conducting miRNA-mRNA correlation analysis and target analysis, we discovered 76 miRNA-mRNA interaction pairs involving 18 miRNAs and 30 mRNAs. Notably, *PPARD* was targeted by gga-miR-130b-3p, gga-miR-146c-3p, and gga-miR-454-3p, while LPL was targeted by gga-let-7j-3p, gga-miR-200b-3p, and gga-miR-29b-3p. It is worth mentioning that *FABP5* and *IGFB3* were exclusively targeted by gga-miR-122-5p (Fig. 2C). Based on the PPI and miRNA-mRNA regulatory networks, we found that *FABP5* was an essential gene involved in these networks. We hypothesize that it plays a crucial role in the development of abdominal fat.

### The FABP5 was targeted by miR‑122‑5p

**Fig. 3:**
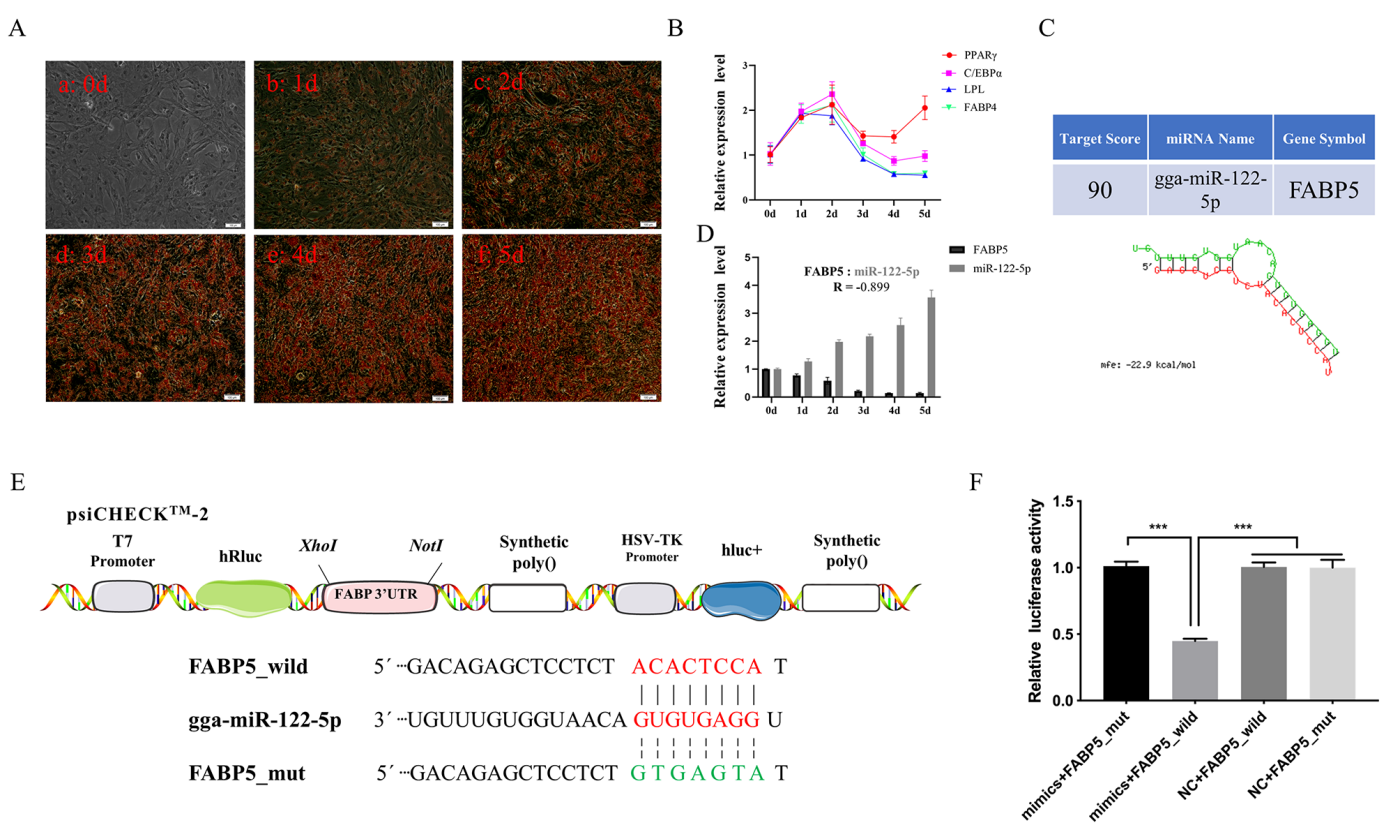
Verification of the target relationship between miR-122-5p and *FABP5*. (**A**) Oil red O staining of preadipocytes during differentiation of chicken abdominal preadipocytes, (a) representative before differentiation of preadipocytes, b, c, d, e and f represent the differentiation after 1, 2, 3, 4, 5 days. (**B**) Relative expression level of adipocyte differentiation marker gene in chicken abdominal preadipocyte differentiation. (**C**) *FABP5* was predicted to be a target of miR-122-5p by miRDB and RNAhybrid software. (**D**) Expression of miR-122-5p and *FABP5* during chicken abdominal preadipocyte differentiation. (**E**) Binding site of miR-122-5p and the 3’UTR sequence of *FABP5* gene. (**F**) The dual-luciferase reporter system assessed the binding of FABP5 and miR-122-5p in DF-1 cells. The data are expressed as the mean ± S.E.M. (* *p < 0.05*; ** *p < 0.01*, *** *p < 0.001*)

To account for the dynamic nature of adipocyte development, we established a model of preadipocyte-induced differentiation. We isolated abdominal preadipocytes and induced their differentiation for 5 days, which resulted in the complete differentiation of adipocytes and production of large lipid droplets (Fig. 3A). Furthermore, we detected the expression levels of adipocyte differentiation markers, including peroxisome promoter activated receptor γ (*PPAR γ*), CCAAT/enhancer binding protein α (*C/EBPα*), lipoprotein lipase (*LPL*), and fatty acid binding protein 4 (*FABP4*) (Fig. 3B). The results showed that the expression of PPAR γ peaked on the second day after induction of differentiation, followed by a decrease and then an increase on Day 5. The expression pattern of *C/EBPα*, *LPL*, and *FABP4* was similar to that of *PPAR γ*; the expression of *LPL* and *FABP4* continued to be downregulated at Days 3, 4, and 5, consistent with previous studies[25]. These results indicated the successful in vitro construction of an induced differentiation model of abdominal preadipocytes, which could be utilized in subsequent experiments. We employed the MiRDB and RNAhybrid bioinformatics prediction software and found that miR-122-5p targets *FABP5* (Fig. 3C). As miRNA expression generally exhibits a negative correlation with mRNA expression, we examined the expression levels of miR-122-5p and *FABP5*. Interestingly, the expression level of miR-122-5p gradually increased along with preadipocyte differentiation, demonstrating a significant 3.5-fold increase at Day 5 compared to proliferating cells (Day 0), while the expression of FABP5 continuously decreased (Fig. 3D). These results indicated that the expression patterns of miR-122-5p and *FABP5* were negatively correlated during preadipocyte differentiation. To confirm that FABP5 was being targeted by miR-122-5p, we conducted a dual-luciferase reporter assay. We found that transfection of the miR-122-5p mimic and FABP5 wild-type plasmids led to a significant reduction in relative luciferase activity compared to the NC group (p < 0.001). However, luciferase activity remained unchanged after co-transfection of FABP5-MUT with the miR-122-5p mimic or NC mimic (Fig. 3E-F). Therefore, our findings suggest that FABP5 is targeted by miR-122-5p.

### MiR-122-5p promotes chicken preadipocyte differentiation by targeting FABP5

**Fig. 4:**
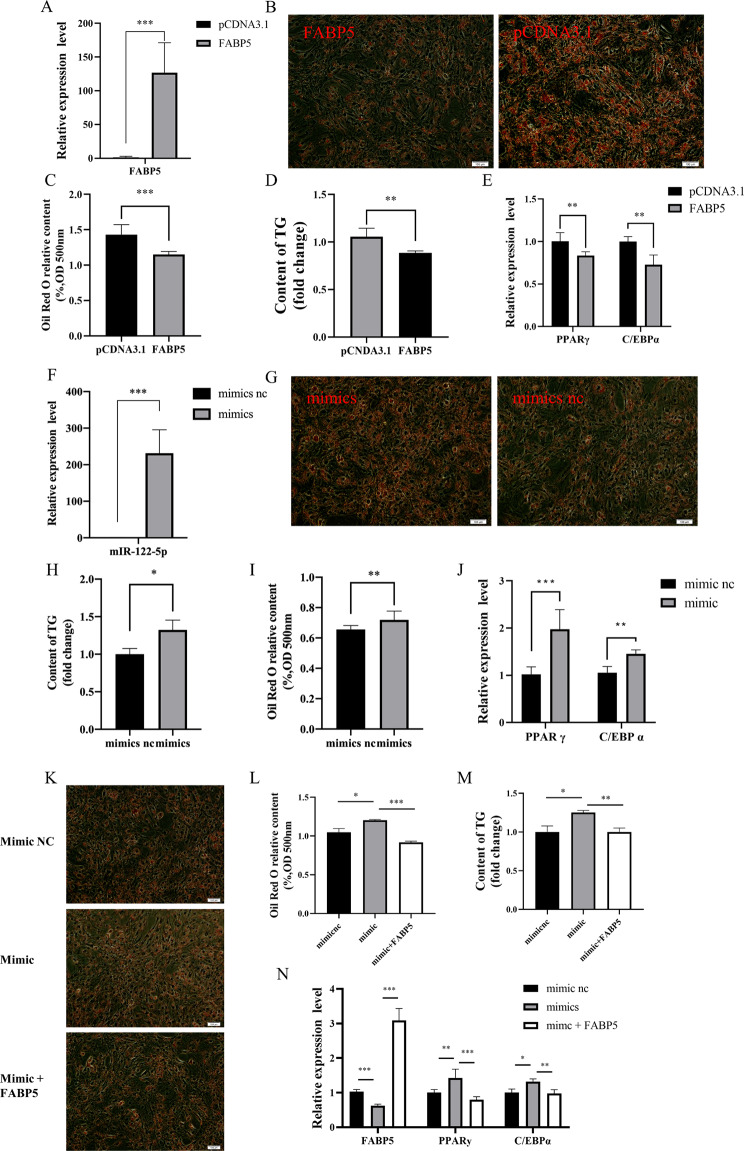
The effect of *FABP5* and miR-122-5p on the differentiation of preadipocytes in chicke.(**A**) Relative expression of *FABP5* after overexpression in preadipocytes. (**B-C**) Oil red O staining results after transfection of *FABP5*. (**D**) Triglycerides concentration of adipocytes of transfection with *FABP5*. (**E**) The relative mRNA level of adipocyte specific genes including *PPAR γ* and *C/EBP α* after transfection with *FABP5*. (**F**) miR-122-5p overexpression efficiency was detected by RT-qPCR. (**G-H**) Oil red O staining results after transfection of miR-122-5p mimics. (**I**) Triglycerides concentration of adipocytes of transfection with miR-122-5p mimics. (**J**) Relative expression of miR-122-5p after miR-122-5p mimics in preadipocytes. (**K-M**) After co-transfection of miR-122-5p and *FABP5* genes, adipocytes were stained with oil red O and detected for triglyceride. (**N**) The mRNA levels of *FABP5*, *PPAR γ*, and *C/EBP α* were expressed after co-transfection of miR-122-5p with *FABP5*. The data are expressed as the mean ± S.E.M. (* *p < 0.05*; ** *p < 0.01*, *** *p < 0.001*)

After confirming the targeting relationship between *FABP5* and miR-122-5p, we further investigated their effects on the differentiation of preadipocytes. To investigate the role of *FABP5* in the differentiation of preadipocytes, we introduced the FABP5 overexpression vector, NC, si-FABP5, or si-NC into chicken abdominal preadipocytes. The expression of *FABP5* was significantly increased in preadipocytes transfected with the *FABP5* overexpression vector, while its expression was significantly suppressed in preadipocytes transfected with si-FABP5 (Fig. 4A, Figure S2A). Furthermore, *FABP5* overexpression inhibited the aggregation of lipid droplets in adipocytes, reduced the accumulation of TG, and suppressed the mRNA expression levels of *PPAR γ* and *C/EBPα* (Fig. 4B-E). In contrast, interference of *FABP5* yielded opposite results (Figure S2B-E). In conclusion, the above results demonstrate that *FABP5* can inhibits the differentiation of preadipocytes. Furthermore, we investigated the potential role of miR-122-5p in the differentiation of preadipocytes. We introduced miR-122-5p mimic, NC mimic, miR-122-5p inhibitor or NC inhibitor into chicken abdominal preadipocytes. The results showed that the expression of miR-122-5p was significantly increased in the preadipocytes transfected with the miR-122-5p mimic and significantly suppressed in the preadipocytes transfected with the miR-122-5p inhibitor (Fig. 4F, Figure S2F). Moreover, overexpression of miR-122-5p increased the aggregation of lipid droplets in adipocytes, increased the accumulation of TG, and upregulated the mRNA expression levels of *PPAR γ* and *C/EBPα* (Fig. 4G-J). In contrast, inhibition of miR-122-5p significantly decreased these detection indicators (Figure S2G-J).

Based on the aforementioned results, it appears that *FABP5* and miR-122-5p exhibit opposing trends in the differentiation of preadipocytes. As such, in order to investigate whether miR-122-5p regulates chicken abdominal preadipocytes through *FABP5*, the miR-122-5p mimic vector, NC mimic vector and miR-122-5p mimic vector for FABP5 overexpression were respectively transfected into chicken abdominal preadipocytes. The results showed that the lipid droplet and TG contents in the miR-122-5p mimic + FABP5 cotreatment group were significantly lower than those in the miR-122-5p mimic group (*P < 0.001*) (Fig. 4K-M). Furthermore, qRT–PCR analysis indicated that compared to the miR-122-5p mimic group, the miR-122-5p mimic + FABP5 cotreatment upregulated the expression of *FABP5* mRNA, while the mRNA expression levels of *PPAR γ* and *C/EBPα* were significantly downregulated (Fig. 4N). Taken together, these results demonstrated that miR-122-5p promotes the differentiation of chicken preadipocytes by directly targeting *FABP5*.

## Discussion

As we are well aware, the process of abdominal fat deposition in chickens is highly complex and continuous, regulated by a diverse array of regulatory factors. In order to identify the pivotal genes and pathways involved in the development of abdominal fat in chickens, transcriptomic data of abdominal adipose tissue at 6, 14, 22, and 30 weeks were analyzed to identify genes that may play key roles at different developmental stages and their potential signaling pathways. Through association analysis with miRNA transcriptome, it was discovered that *FABP5* may be regulated by miR-122-5p and thus affect the deposition of abdominal fat. Cell experiments confirmed that miR-122-5p upregulated the expression of *PPAR γ* and *C/EBP α* differentiation marker genes by inhibiting *FABP5*, leading to increased accumulation of triglycerides in adipocytes and promoting the formation of lipid droplets. In summary, these findings not only enhance our understanding of the intricacies of abdominal fat deposition in chickens, but also identify novel markers and targets for improving meat quality.

In this study, a strict screening process yielded a total of 1893 DEGs. Time cluster analysis of DEGs revealed a downward trend in gene expression for cluster1 during development, while an upward trend was observed for cluster4. Functional enrichment analysis indicated significant enrichment of the TGF-β signaling pathway and Wnt signaling pathway in cluster1. These two pathways play crucial roles in regulating gene transcription and affecting cell proliferation and differentiation[26–28]. *TGFB3* has an inhibitory effect on adipocyte generation and lipid droplet aggregation, whereas c*MYC* activation stimulates the proliferation of 3T3 preadipocytes[29–31]. In this study, both actors participating in these pathways have gradually decreased in expression levels. This substantiates the notion that the TGF-β signaling pathway and Wnt signaling pathway regulate the development of abdominal fat at about 6 weeks. Moreover, the PPAR signaling pathway was also enriched in cluster1, comprising *FABP1*, *FABP5*, *FABP7*, *ADIPOQ*, and *ACOX1*, among others. *ACOX1* plays a role in promoting fatty acid oxidation and reducing the differentiation of preadipocytes[32]. *ADIPOQ* increases the activities and expressions of enzymes that promote triglyceride hydrolysis and fatty acid breakdown[33]. These findings suggest that the development of abdominal fat before 6 weeks is driven by the proliferation and differentiation of preadipocytes, rather than fat deposition. On the other hand, Cluster 2 and 3 showed a significant enrichment in the Drug metabolism - other enzymes and Drug metabolism - cytochrome P450 pathways. Cytochrome P450 was involved in the oxidation of steroids and fatty acids, and in regulating the activation, deactivation, and conversion of steroid hormones[34]. The metabolism of steroid hormones in adipose tissues was significantly disrupted during obesity. This indicates that at 14 and 20 weeks, the main function of chicken abdominal fat tissue was to regulate steroid hormones. In contrast, expression of genes in cluster4 was up-regulated with development. Enriched signal pathways included apoptosis and the PPAR signaling pathway, which includes *ACSl4*, *ACSL6*, *LPL*, and *PLIN2*. These genes are highly correlated with fatty acid metabolism and lipid droplet aggregation[35–37]. During the peak egg production period in chickens at 30 weeks, adipocyte apoptosis provides energy for egg production, while excess dietary nutrients are converted into triglycerides and stored in adipocytes for the body’s needs, which is in accordance with previous research findings[24]. Xiao et al. observed that the PPAR signaling pathway and Wnt signaling pathway were associated with fat deposition in Nandan Yao chickens[11]. Additionally, Wang et al. found a correlation between the PPAR signaling pathway, fatty acid metabolism, and fatty acid oxidation with fat deposition in broiler chickens, which is consistent with the findings of our research[38].

The deposition of abdominal fat involves the generation of new adipocytes and the accumulation of triglycerides within adipocytes [39]. The regulation of key genes is crucial for this physiological process, in which miRNAs play an essential role in post-transcriptional regulation [40]. In this study, we identified a set of genes closely associated with the deposition of abdominal fat. Through miRNA-mRNA correlation analysis, we constructed an interaction network between miRNAs and mRNAs associated with adipose development. In these interaction networks, *PPARD* was identified as a potential target of gga-miR-130b-3p, gga-miR-146c-3p, and gga-miR-454-3p. It has been found that miR-130b may target *PPAR γ*, inhibiting adipose deposition in mouse epididymal fat pads [41]. miR-146 can accelerate the lipolysis of white adipose tissue [42], while miR-454 can target *PPAR γ* and inhibit triglyceride synthesis in bovine epithelial cells[43]. Interestingly, we found that *FABP5* is only targeted by gga-miR-122-5p, which has been shown to play an active role in hepatic lipid metabolism [44]. The results of the PPI analysis suggest the formation of a highly complex regulatory network among these genes. *PPARD*, *ACOX1*, and *FASN* serve as central nodes connecting genes involved in fatty acid transport and metabolism. Numerous studies have demonstrated the strong correlation between *FASN* and fatty acid content, and its ability to alter fatty acid content. *FASN* has been identified as a key enzyme involved in mouse adipogenesis. Notably, *FABP5* is closely linked to *FASN* in this network. It is noteworthy that the involvement of *FABP5* in the development of abdominal fat in chickens has caught our attention.

During preadipocyte differentiation, we found that the expression of *FABP5* was continuously downregulated and negatively correlated with the expression of miR-122-5p. As a member of the fatty acid transporter family, *FABP5* plays an important role in fatty acid absorption, transport and metabolism. Therefore, we hypothesized that *FABP5* may be involved in chicken abdominal adipogenesis and may be regulated by miR-122-5p. To test our hypothesis, we overexpressed *FABP5* in preadipocytes and found that *PPARγ* and *C/EBPα* mRNA levels were downregulated, which reduced the triglyceride content and inhibited the generation of lipid droplets. *FABP5* interference showed the opposite results. Studies have found that the high expression of *FABP5* in cancer cells promotes lipolysis, and the lipolysis of adipocytes in *FABP5* transgenic mice is increased. Knockdown of *FABP5* promotes the accumulation of triglycerides in retinal pigment epithelial (RPE) cells, which was consistent with our results [45–47]. Interestingly, miR-122-5p and *FABP5* had opposite effects on the differentiation of chicken abdominal preadipocytes. In particular, the expression of *FABP5* was decreased significantly by miR-122-5p overexpression in chicken abdominal preadipocytes. However, miR-122-5p interference resulted in upregulated *FABP5* expression. Dual-luciferase reporter assays indicated that miR-122-5p directly targeted *FABP5*. To determine whether miR-122-5p plays a key role in the differentiation of preadipocytes through *FABP5*, we performed rescue experiments. The results showed that co-transfection of miR-122-5p and *FABP5* reversed the promotion of preadipocyte differentiation by miR-122-5p and inhibited the expression of *PPAR γ* and *C/EBPα*. These results, along with those from previous studies, indicated that the role of miR-122-5p in chicken abdominal preadipocytes is mainly to reduce lipolysis by inhibiting the expression of *FABP5*, thereby promoting abdominal fat deposition in chickens.

The PPAR signaling pathway has a wide range of biological functions in animals and regulates important physiological processes, such as lipid metabolism, energy balance, cell division and cell differentiation [48, 49]. Among them, *PPAR γ* has been found to activate preadipocyte differentiation into adipocytes and to be the main regulator of adipogenesis [50, 51]. In addition, *PPARγ* is activated by fatty acids and exogenous peroxisome proliferators, thereby regulating the differentiation of preadipocytes [52]. Of note, *FABP5*, which was shown to be directly targeted by miR-122-5p in the present study, is an upstream gene of PPARs. In the PPAR signaling pathway, *FABP5* may regulate the activation of *PPAR γ*. Some studies have found that *FABP5* overexpression increases lipolysis in adipocytes and that *FABP5* and *FABP4* have mutual compensatory effects on adipocyte lipolysis​ [45, 53]. In mouse preadipocytes, *FABP4* triggers the ubiquitination and subsequent proteasomal degradation of *PPAR γ*, thereby inhibiting the accumulation of lipid droplets [54]. The present study confirmed that miR-122-5p promotes the differentiation of preadipocytes by inhibiting *FABP5*. Based on the above results and those of a previous study, we propose the following underlying mechanism: (1) miR-122-5p targets *FABP5* and inhibits its expression, which reduces the effect of the FABP family on *PPARγ* ubiquitination; and (2) *PPARγ* expression is upregulated, which promotes the differentiation of preadipocytes, resulting in the accumulation of lipid droplets in abdominal adipocytes.

## Conclusions

In conclusion, we described the transcriptome expression profile of abdominal adipose tissue in Gushi chicken at 6, 14, 22 and 30 weeks of development, and identified the cluster highly related to abdominal adipose deposition through time series analysis. The construction of PPI and miRNA-mRNA network also showed the complexity of abdominal adipose deposition. In addition, miR-122-5p is a positive regulator of abdominal preadipocyte differentiation in Gushi chicken, while *FABP5* is a negative regulator of abdominal preadipocyte differentiation in Gushi chicken. Target verification and rescue experiment showed that miR-122-5p directly targeted *FABP5* and down-regulated its expression, thus promoting the differentiation of abdominal preadipocytes and the accumulation of lipid droplets in Gushi chicken. The results provide a new insight into the mechanism of abdominal fat deposition in chickens.

## Methods

### Transcriptome library construction and sequencing

The present investigation involved the use of Chinese Gushi chickens as experimental animals. The experimental animals in this study were Gushi chickens from the Animal Center of Henan Agricultural University. A total of 200 one-day-old female Gushi chickens were raised in cages with the same environment, with standard conditions for pure breeding conservation of Gushi chickens. In this study, three healthy chickens were randomly selected at 6, 14, 22, and 30 weeks of age. Therefore, twelve chickens were used in this study. Our sample size was sufficient, and the remaining healthy chickens are still used for the pure breeding conservation of Gushi chickens. These chickens had a two-stage feeding protocol, in which 18.5% crude protein and 12.35 MJ/kg were prepared in the first stage (younger than 14 weeks) and 15.6% crude protein and 12.75 MJ/kg were prepared in the second stage (older than 14 weeks), and the chickens had free access to water. The chickens were anesthetized by an intravenous injection of sodium pentobarbital (40 mg/kg) at a concentration of 0.2% into the wing vein. Under deep anesthesia, the chickens were euthanized by an intravenous injection of KCl (1–2 mg/kg), after which their abdominal adipose tissues were harvested, immediately frozen in liquid nitrogen and stored at − 80 °C until total RNA extraction. The Trizol method was employed to extract the total RNA of the abdominal adipose tissue, in strict accordance with the operation instructions of the RNA isolater Total RNA Extraction Reagent (Vazyme, China) kit. The degradation level and contamination of RNA were evaluated through 1% agarose gel electrophoresis, while the concentration and purity of RNA were determined by ultraviolet spectrophotometry. The RNA samples exhibiting a 260 and 280 ratio were selected. The samples that qualified after quality inspection were purified by the RNA Clean XP Kit (Beckman Coulter, America) and the RNase-Free DNase Set (QIAGEN, Germany).

After eliminating the rRNA from the total RNA sample, a short 250-300 bp segment was used as a template to synthesize the first-strand cDNA, followed by the synthesis of double-stranded cDNA. An A-tail was added, and the sequencing connector was linked. The chain-specific cDNA library was ultimately produced through PCR amplification and enrichment. The initial insert size of the constructed library was detected using Agilent 2100, and after the test was found to be qualified, the effective concentration of the library was adjusted to > 2 nM through the q-PCR method. Lastly, RNA sequencing was carried out using Illumina HiSeqTM 2500 (novogene, china).

### Quality control and analysis of the sequencing data

Initially, the raw sequencing data (raw reads) was processed using the fastp software [55], in which adapter sequences, polymeric reads, and low-quality reads were removed to obtain pure data (clean reads). Concurrently, the Q20, Q30, and GC content of the clean data were calculated. The reference genome and gene annotation file for the chicken were downloaded from the genome website (https://ftp.ensembl.org/pub/release-104/fasta/gallus_gallus/dna/). The reference genome index was constructed using Hisat2, and the paired-end reads were aligned to the reference genome [56]. The featurecounts software was utilized to calculate the read counts of mRNAs in each sample[57]. StringTie was used to calculate the FPKM (Fragments Per Kilobase of exon model per Million mapped fragments) of mRNAs in each sample, and the FPKM value was used to evaluate the expression level of mRNAs in each library[56].

### Differential expression analysis and clustering analysis of the time series

The DEseq2 R package was used for differential expression analysis of 6 comparison groups (W14 vs. W6, W22 vs. W14, W30 vs. W22, W22 vs. W6, W30 vs. W6, and W30 vs. W14), with a threshold standard of corrected p-value (q-value) < 0.05 and log2FoldChange > |1| used for screening significant differentially expressed mRNAs (DE-mRNAs) [58].To identify dynamic gene expression changes during the development of abdominal fat in Gushi chickens, the time series clustering analysis was performed using the Mfuzz R package on the union of DE-mRNAs from all comparison groups based on the FPKM values in each sample [59]. For this purpose, the log2-fold change of differentially expressed genes was calculated at different stages of abdominal fat development in Gushi chickens, and clustering of the differentially expressed genes was performed in Euclidean space.

### Enrichment analysis of GO and KEGG pathways

The DE-mRNAs were subjected to GO term and KEGG pathway enrichment analyses using the clusterProfiler 4.0 R package[60, 61]. Enriched GO terms and KEGG signaling pathways with a *q < 0.05* were considered significant.

### Analysis of associations and construction of interactive networks

In addition to mRNA transcriptome data, we also constructed the miRNA transcriptome profile related to abdominal fat development in Gushi chickens using the same tissue samples [24]. Based on the miRNA transcriptome data, the potential targeting interactions between differentially expressed mRNAs and miRNAs were predicted using miRanda and TargetScan software [62]. Based on the expression levels of mRNAs and miRNAs in the four developmental stages of Gushi chicken abdominal fat, and through Pearson correlation analysis, miRNA-mRNA pairs with negatively correlated expression levels were selected to construct the miRNA-mRNA interaction network. Protein-protein interaction (PPI) analysis was performed using the STRING database[63]. Visualizations were generated using the Cytoscape software (http://www.cytoscape.org/).

### Preadipocyte isolation and induced differentiation

Chicken abdominal preadipocytes were separated according to a previous method [64]. Briefly, under aseptic conditions, the abdominal adipose tissue of 14-day-old Gushi chickens was washed 3 times in phosphate buffer (PBS) (Solarbio, China) containing 0.8% streptomycin/penicillin. The tissue was then cut into 2 mm pieces and digested in serum-free DMEM/F12 (BI, Israel) containing 2 mg/ml collagenase-I (Solarbio, China) and 150 mg/mL bovine serum albumin (BSA) (Solarbio, China) for 90 min and oscillated once every 5 min. Digestion was terminated by adding culture medium, and the cell suspension was filtrated through 150 μm, 74 μm, 25 μm, respectively. The filtrate was centrifuged at 1500 rpm for 10 min, and the cell precipitate was washed twice with complete culture medium. The cell precipitate was resuspended at a density of 5 × 10^4^ cells/ml in DMEM/F12 medium (Bi, Israel) containing 10% fetal bovine serum (BI, Israel) and 0.1% penicillin/streptomycin (Solarbio, China) followed by culture at 37 °C in a 5% CO_2_ incubator. According to the manufacturer’s instructions, miRNA and siRNA were transfected with Lipofectamine 3000 reagent (Invitrogen, California, USA). Chicken abdominal preadipocytes were seeded into 6-well plates at a density of 2 ~ 3 × 10^5^ cells per well in DMEM/F12 medium (Bi, Israel) containing 10% fetal bovine serum and 160 µM sodium oleate (Sigma, Germany) [25]. The medium was changed every day until the fifth day of culture, and cells were collected at 0, 1, 2, 3, 4 and 5 days. The differentiation of adipocytes was observed daily using microscopy and oil red O staining.

### RNA extraction, cDNA synthesis and quantitative real-time PCR (qRT–PCR)

Total RNA was extracted from cells using TRIzol reagent (Vazyme, China), and quantitative determination of RNA concentration was performed by spectrophotometry (Thermo, Waltham, MA, USA). Total RNA was reverse transcribed into cDNA using HiScript II Q RT SuperMix for qRT–PCR (+ gDNA wiper) (Vazyme, Nanjing, China). qRT–PCR was performed on a LightCycler® 96 qRT–PCR system (Roche, Basel, Switzerland) with ChamQ Universal SYBR qPCR Master Mix (Vazyme Biotech, Nanjing, China). *β-actin* was used as an internal reference gene for mRNA, and *U6* was used as the internal reference gene for miRNA. The relative expression levels were analyzed with the 2^−ΔΔCt^ method. The experiments were performed with a minimum of three technical replicates, and all data are presented as the mean ± standard error of the mean. The primer sequences are listed in Table S2.

### Plasmid construction and transfection

The chicken *FABP5*-coding region was cloned using the *FABP5* primer (CDS) and cleaved using EcolΙ and HindIII, and the fragment was then inserted into pcDNA3.1-EGFP to obtain pcDNA3.1-EGFP-FABP5 (Table S2). Similarly, pcDNA3.1-EGFP was used as a negative control (NC). An inhibitor of *FABP5* (si-FABP5; sense, GCUUUAAAGUGCCACAAUATT; and antisense, UAUUGUGGCACUUUAAAGCTT) and NC (si-NC) were designed by GenePharma (Shanghai, China). The 3’UTR fragment of *FABP5* containing the binding sites was amplified by PCR from cDNA and then cloned into a psiCHECKTM-2 dual-luciferase reporter vector. To construct the mutant vectors, we designed a mutant primer sequence and changed the binding site (Table S2). Eight binding sequences were successfully mutated from ACACTCC to GTGAGTA to obtain the mutant FABP5-3’UTR vector. The BµLge-Loop™ miRNA qRT–PCR-specific mimic (sense, UGGAGUGUGACAAUGGUGUUUUGU; and antisense, AAACACCAUUGUCACACUCCAUU) and inhibitor (ACAAACACCAUUGUCACACUCCA) primers were designed by GenePharma (Shanghai, China). Chicken abdominal preadipocytes were seeded in 6-well plates and transfected when they reached 80% confluence. Cells were transfected with miR-122-5p mimics, negative control (NC), pcDNA3.1-EGFP-FABP5 and pcDNA3.1-EGFP using Lipofectamine 3000 (Invitrogen, Carlsbad, CA, USA). In addition, abdominal preadipocytes were induced to differentiate after 24 h of transfection, and cells were collected after 24 h of induction to evaluate the changes in relevant indicators.

### Target gene prediction and dual-luciferase reporter assays

The target genes of miRNAs were predicted by miRDB and RNAhybrid bioinformatics prediction software [65, 66]. The miRNA target verification assay was performed using DF-1 cells [67]. Wild-type or mutant *FABP5*-3’UTR dual-luciferase reporter vectors and miR-122-5p mimics or NC duplexes were co-transfected into DF-1 cells in 24-well plates using Lipofectamine 3000 reagent (Invitrogen, California, USA).

### Oil red O staining and quantification

Cells were inoculated into 6-well plates. After 48 h of transfection, cells were washed three times with PBS buffer, fixed with 4% formaldehyde for 40 min, washed with PBS and stained with 0.5% oil red O for 10 min. The oil red O stain was then removed, and cells were washed three times with PBS. Subsequently, stained cells were visualized using a fluorescence microscope (Olympus, Japan), and images were acquired for analysis. Finally, a quantitative assessment was obtained by spectrophotometric analysis of the absorbance at 500 nm.

### Triglyceride content assay

The triglyceride (TG) concentrations in cell lysates were determined by a triglyceride assay kit (APLLYGEN, Beijing, China). The TG concentrations were normalized to the protein content using the BCA Protein Detection Kit (Bioteke Corporation, Beijing, China) according to the manufacturer’s protocol for TG content analysis.

### Statistical analysis

All experiments were performed in triplicate, and the data are expressed as the mean ± S.E.M. A t test was used to determine statistically significant differences between two groups (* *p < 0.05*, ** *p < 0.01* and *** *p < 0.001*). GraphPad Prism 8.0 software (San Diego, CA, USA) was used for the statistical analysis and data visualization.

## Electronic supplementary material

Below is the link to the electronic supplementary material.

Supplementary Material 1

Supplementary Material 2

Supplementary Material 3

Supplementary Material 4

## Data Availability

The authors declare that the data supporting the findings of this study are available within the article and its supplementary information files. All the raw sequences have been deposited in the NCBI database Sequence Read Archive with the accession numbers SRR9610426–SRR9610437 (BioProject number PRJNA551368).
