# Supplementary material for: Transcriptome analysis reveals FABP5 as a key player in the development of chicken abdominal fat, regulated by miR-122-5p targeting
Source: BMC Genomics. 2023 Jul 10;24:386. doi: 10.1186/s12864-023-09476-1 (PMC10331962; doi:10.1186/s12864-023-09476-1)
Supplement: Supplementary file 1 — Supplementary Material 1 [file 12864_2023_9476_MOESM1_ESM.docx]

**Table S1. Summary of draft reads of 12 cDNA libraries, determined by RNA- sequencing.**

| **Sample** | **Raw_reads** | **Clean_bases** | **Error_rate(%)** | **Q20(%)** | **Q30(%)** | **GC_content(%)** | **Total mapped(%)** |
| --- | --- | --- | --- | --- | --- | --- | --- |
| W6_1 | 93,729,158 | 13.53GB | 3.65 | 97.88 | 94.10 | 47.86 | 95.49 |
| W6_2 | 105,587,052 | 15.27GB | 3.43 | 97.95 | 94.24 | 48.50 | 92.96 |
| W6_3 | 105,361,016 | 15.24GB | 3.41 | 97.94 | 94.24 | 50.05 | 94.30 |
| W14_1 | 111,668,572 | 16.02GB | 4.22 | 97.39 | 92.68 | 48.69 | 94.62 |
| W14_2 | 99,103,024 | 14.17GB | 4.43 | 97.37 | 92.65 | 46.20 | 95.04 |
| W14_3 | 107,542,426 | 15.37GB | 4.61 | 97.31 | 92.47 | 48.97 | 93.95 |
| W22_1 | 111,838,654 | 16.21GB | 3.33 | 97.92 | 94.19 | 51.52 | 94.71 |
| W22_2 | 116,176,278 | 16.86GB | 3.11 | 97.94 | 94.23 | 51.56 | 94.00 |
| W22_3 | 97,137,262 | 13.78GB | 5.31 | 97.15 | 92.11 | 47.87 | 95.59 |
| W30_1 | 113,865,916 | 16.45GB | 3.48 | 97.75 | 93.75 | 48.90 | 91.97 |
| W30_2 | 108,976,258 | 15.74GB | 3.5 | 97.75 | 93.75 | 47.94 | 93.30 |
| W30_3 | 116,860,112 | 16.88GB | 3.53 | 97.75 | 93.76 | 47.63 | 94.54 |

Abbreviations: W6_1, sample 1 of 6 weeks; W6_2, sample 2 of 6 weeks; W6_3, sample 3 of 6 weeks; W14_1, sample 1 of 14 weeks; W14_2, sample 2 of 14 weeks; W14_3, sample 3 of 14 weeks; W22_1, sample 1 of 22 weeks; W22_2, sample 2 of 22 weeks; W22_3, sample 3 of 22 weeks; W30_1, sample 1 of 30 weeks; W30_2, sample 2 of 30 weeks; W30_3, sample 3 of 30 weeks.
