# Supplementary material for: Transcriptome analysis reveals FABP5 as a key player in the development of chicken abdominal fat, regulated by miR-122-5p targeting
Source: BMC Genomics. 2023 Jul 10;24:386. doi: 10.1186/s12864-023-09476-1 (PMC10331962; doi:10.1186/s12864-023-09476-1)
Supplement: Supplementary file 2 — Supplementary Material 2 [file 12864_2023_9476_MOESM2_ESM.docx]

**Table S2 Primer information used for the qRT-PCR analysis**

| **NCBI Reference Sequence** | **Target** | **Primer sequence (5′-3′)** | **AT**  **(°C)** | **length (bp)** |
| --- | --- | --- | --- | --- |
| NM_204728.2 | *PPARD*(qRT-PCR) | F：ATAACGCAATCCGCTTTGGC  R：CCGGTCAAGATACCTCTCGC | 60 | 190 |
| NM_001031420.2 | *PLIN2*(qRT-PCR) | F：GATCGGCATCCTCCTTCCAG  R：GGACCTACCAGCCAGTTGAG | 60 | 139 |
| NM_001004384.3 | *IGF1*(qRT-PCR) | F：TGGCCTGTGTTTGCTTACCT  R：TCCCTTGTGGTGTAAGCGTC | 60 | 160 |
| NM_001006346.2 | *FABP5*(qRT-PCR) | F：ATGGCCATCGACGCGTT  R：TGCCTTCTGGTAGACTCTAACAC | 60 | 135 |
| NM_205518.2 | *β-actin* | F：CACGGTATTGTCACCAACTG  R：ACAGCCTGGATGGCTACATA | 60 | 200 |
| NM_001006346.2 | *FABP5_*wild | F: CCGCTCGAGGAGCTCCTCTACACTCCATAAT  R:CCGCTCGAGGAGCTCCTCTGTGAGTAATAATTTCCTTTTCC | 62 | 337 |
| NM_001006346.2 | *FABP5_*mut | F:CCGCTCGAGGAGCTCCTCTGTGAGTAATAATTTCCTTTTCC  R:ATAAGAATGCGGCCGCATTAAAGATGAAAAGGCTGGTT | 62 | 337 |
| NM_001006346.2 | *FABP5* (CDS) | F: CCCAAGCTTATGGCCATCGACGCGTT  R: TCCCCGCGGTGCCTTCTGGTAGACTCTAACAC | 59 | 420 |
|  | miR-122-5p(RT) | GTCGTATCCAGTGCAGGGTCCGAGG  TATTCGCACTGGATACGACACAAAC |  |  |
|  | miR-122-5p(qRT-PCR) | F: GCGTGGAGTGTGACAATGGT  R: AGTGCAGGGTCCGAGGTATT | 60 | 67 |
| NM_001302170.2 | *U6* (RT) | GTCGTATCCAGTGCAGGGTCCGAGGTA  TTCGCACTGGATACGACCGATACA |  |  |
| NM_001302170.2 | *U6* (qRT-PCR) | F: GGGCCATGCTAATCTTCTCTGTATCG  R: GTGCAGGGTCCGAGGT |  |  |
| NM_001001460.2 | *PPARγ* (qRT-PCR) | F: GTGCAATCAAAATGGAGCC  R: CTTACAACCTTCACATGCAT | 60 | 170 |
| NM_001031459.2 | *C/EBPα* (qRT-PCR) | F: TTCTACGAGGTCGATTCCCG  R: AGCCTCTCTGTAGCCGTAG | 60 | 96 |
| NM_205282.2 | *LPL* (qRT-PCR) | F: GTACAGTCTGGGTGCTCAT  R: GGAAACCTCCACCATTAG | 60 | 248 |
| NM_204290.2 | *FABP4* (qRT-PCR) | F: ATGTGCGACCAGTTTGT  R: TCACCATTGATGCTGATAG | 60 | 143 |

Abbreviation: AT refers to the annealing temperature; RT refer to the reverse transcription primer sequence; F and R refer to the forward and reverse primers, respectively; Length refers to the length of the product.
