# Supplementary figures and images for: Transcriptome analysis reveals FABP5 as a key player in the development of chicken abdominal fat, regulated by miR-122-5p targeting
Source: BMC Genomics. 2023 Jul 10;24:386. doi: 10.1186/s12864-023-09476-1 (PMC10331962; doi:10.1186/s12864-023-09476-1)

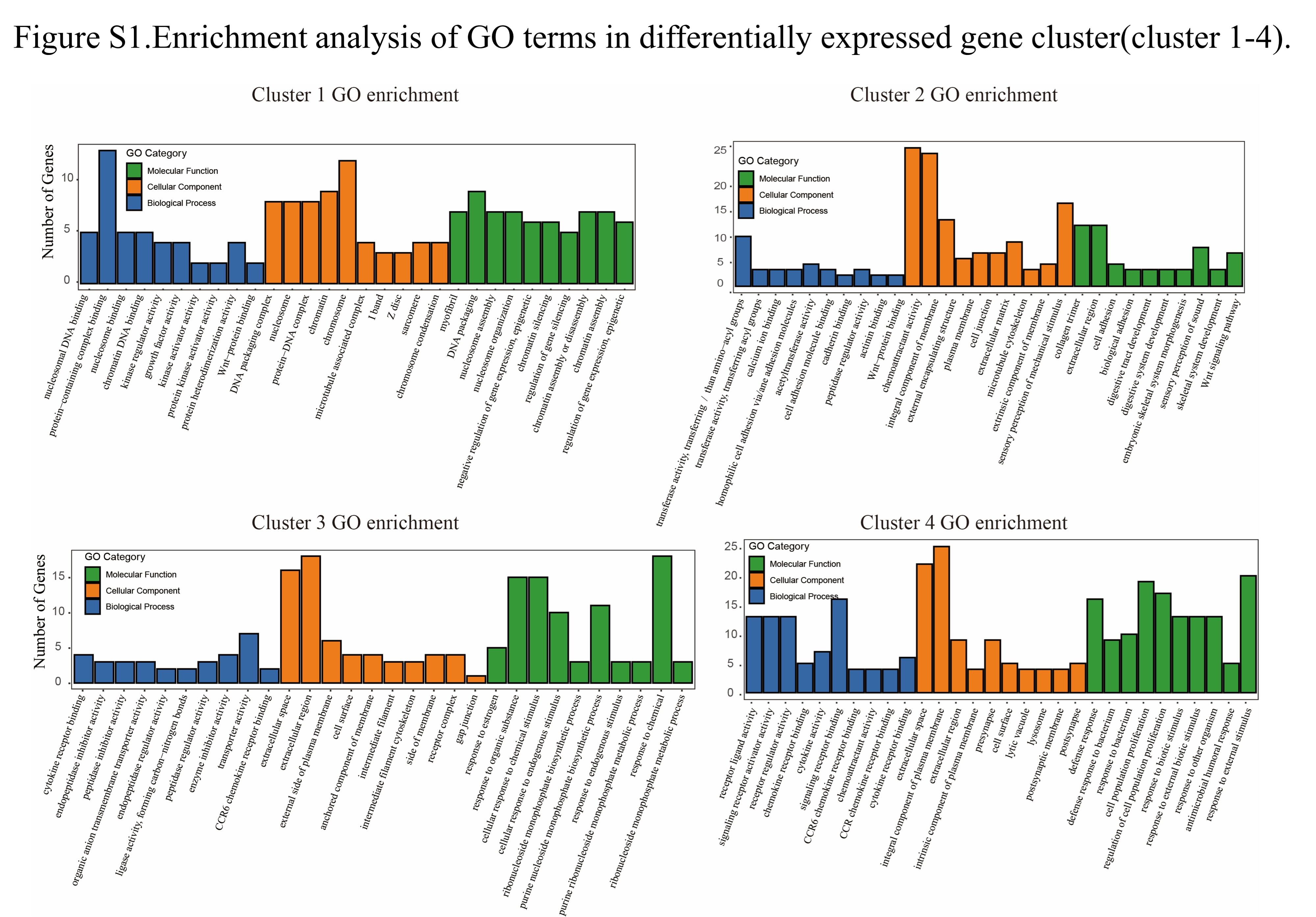

Supplement: Supplementary file 3 — Supplementary Material 3 [file 12864_2023_9476_MOESM3_ESM.tif]

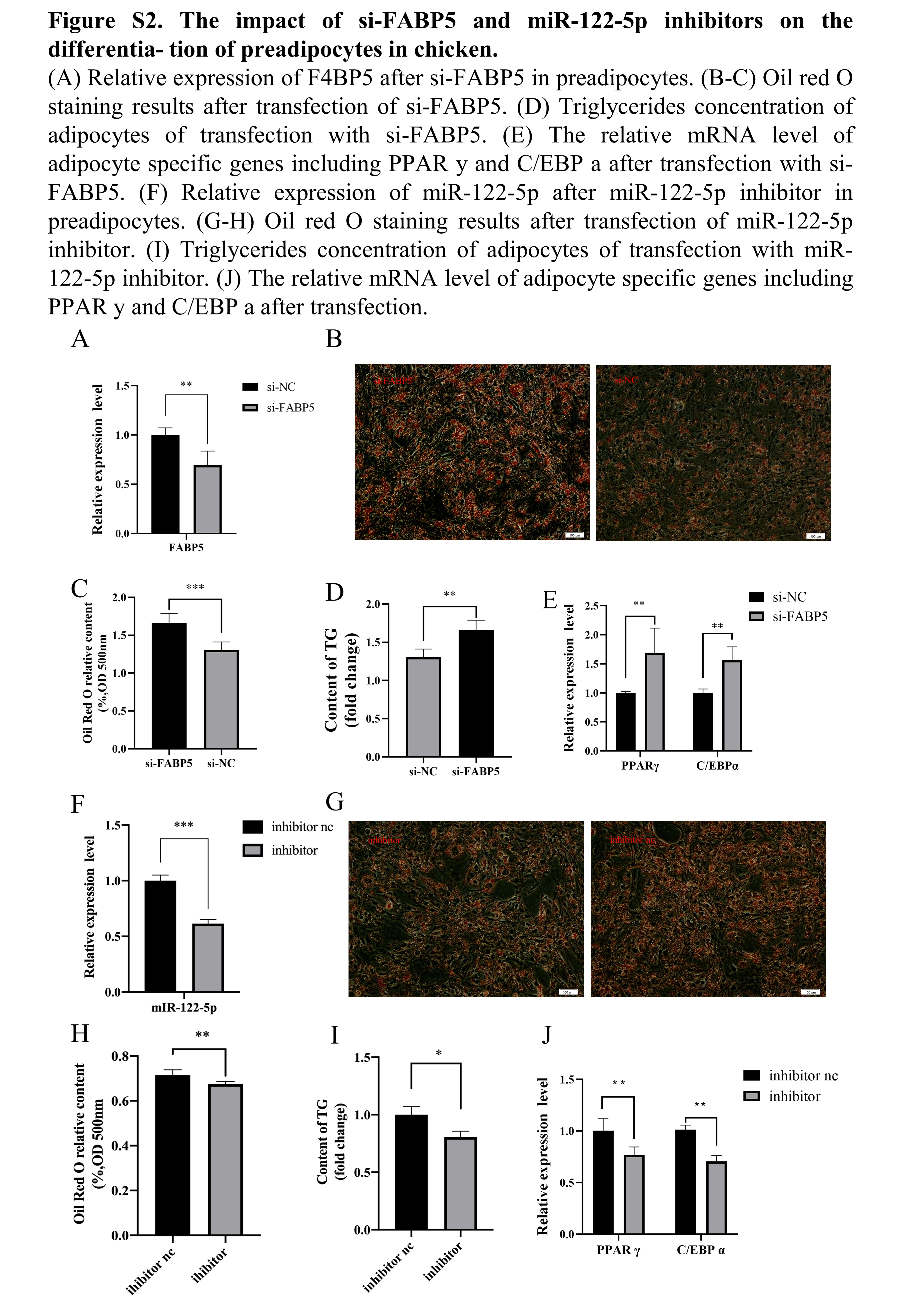

Supplement: Supplementary file 4 — Supplementary Material 4 [file 12864_2023_9476_MOESM4_ESM.tif]
